# A Case of Secondary Syphilis with the Extragenital Chancre on the Nipple

**DOI:** 10.1155/2020/2391907

**Published:** 2020-02-11

**Authors:** Xiao Yan Wang, Juliandri, Zi Jing Liu, Jia Wen Zhang, Yun Yi Liu, Da-Guang Wang, Yang Xu

**Affiliations:** Department of Dermatology, The First Affiliated Hospital of Nanjing Medical University, Nanjing 210029, China

## Abstract

Syphilis is a sexually transmitted disease caused by *Treponema pallidum*. The signs and symptoms of syphilis vary depending on which of the four stages it presents. The primary stage of syphilis classically presents with a painless ulcer (chancre). We report a case of the extragenital chancre on the nipple which is examined from skin biopsy and immunohistochemistry. This case showed that it is important to identify the special site's pruritus erythema by pathology and serological examination.

## 1. Introduction

Syphilis is a common sexually transmitted or blood contracted, congenital transmission disease. It can be divided into primary syphilis, secondary syphilis, tertiary syphilis, and neurosyphilis. The diagnosis is based on the clinical manifestations and serological methods. In this article, we report a case of secondary syphilis with extragenital chancre on the nipple.

## 2. Case Presentation

**Figure 1:**
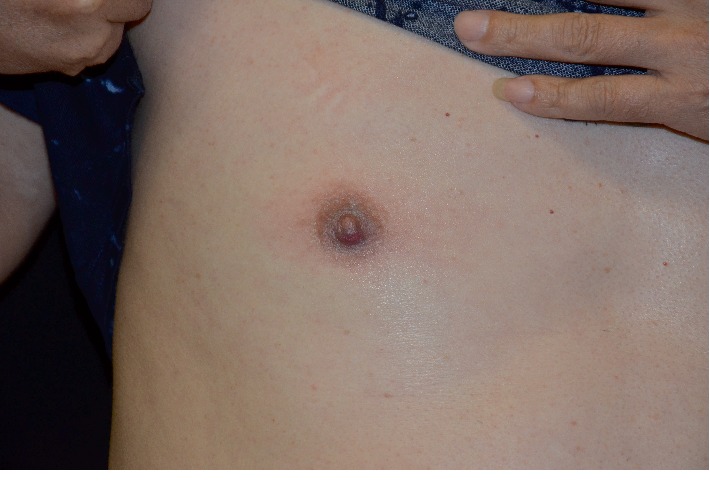
An erythematous erosion with a few exudation on the right nipple before treatment.

**Figure 2:**
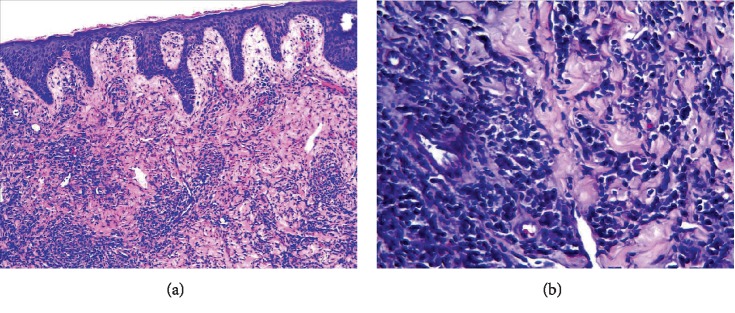
Biopsy of the nipple showed that epidermal hyperplasia, cutaneous protrusion, a migration of small amount of inflammatory cells, an infiltration of large number of cells around the blood vessels of the dermis (a); the inflammatory cells in the dermis were majorly lymphocytes and plasma cells (b). (H-E staining, original magnification: (a) ×100; (b) ×400)

**Figure 3:**
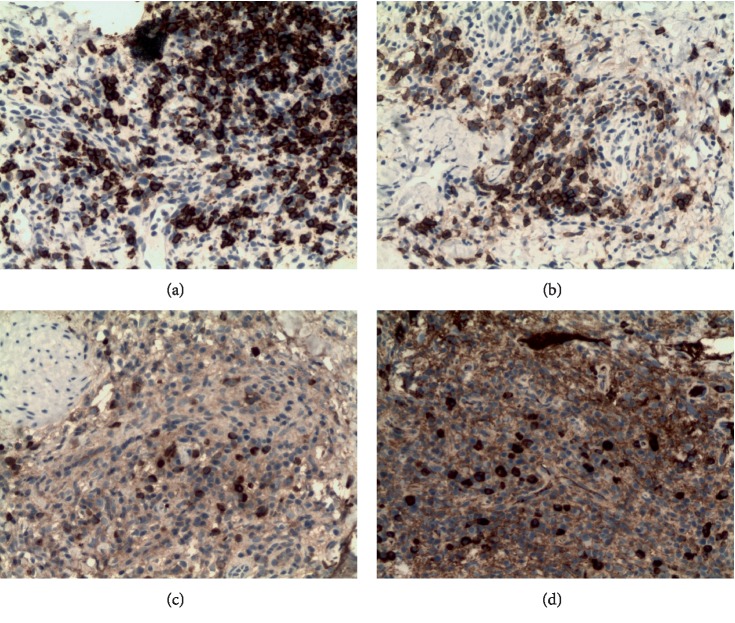
Immunohistochemical staining showed that lymphocplasmocytes infiltration in the cutaneous dermal layer. (a: CD20(+), b: CD38(+), c: lg*κ*(+), d: Ig*λ*(+), original magnification: ×400)

**Figure 4:**
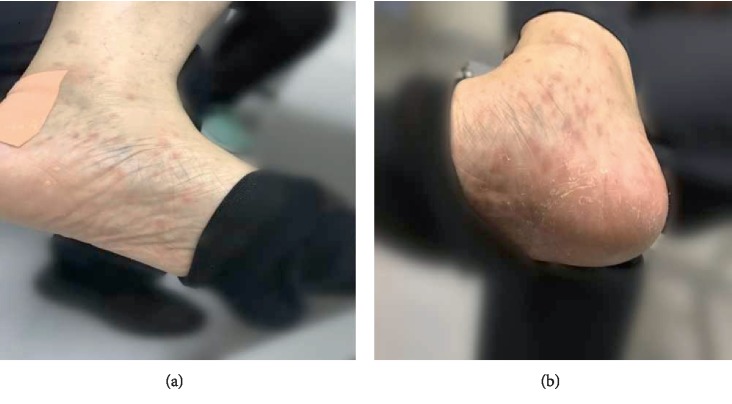
Bilateral palms and soles had multiple copper-red macula and papules some of which were desquamated on the surface. ((a): left sole, (b): right sole).

A 56-year-old male was presented to our dermatology department on June 12, 2018, with complaints of the right nipple erosion and discomfort for a week. Diagnosis of “eczema” was made and the patient was treated with topical zinc oxide oil and traditional Chinese herbal cream. Physical examination showed local erythematous erosion with a few exudations on the right nipple (Figure 1) and a skin biopsy was performed. The histopathological results showed epidermal hyperplasia, cutaneous protrusion, migration of small amount of inflammatory cells, infiltration of large number of cells around the blood vessels of the dermis, and some of the nucleus were large, with scattered cells such as the lymphocytes and plasma cells (Figure 2). Immunohistochemistry was recommended to confirm the diagnosis. The results revealed the (the right papillary skin) proliferation of lymphocytes: CD20(+), CD3(+), CD5(+), Pax-5(+), CD38(+), Bc1-2(+), CD10(−), Bcl-6(−), MUM1(−), Cyclin D1(−), CD163(+), Ig*κ*(+), and Ig*λ*(+), combined with hematoxylin-eosin (H-E) staining, showed numerous lymphoplasmacytes infiltration in the cutaneous dermal layer (Figure 3). On the follow-up, the patient complained of painless itchy erythema on bilateral soles. Physical examination showed that bilateral palms and soles had multiple copper-red macula and papules, some of which were desquamated on the surface (Figure 4). The syphilis serology examination showed that TPPA was positive, RPR was positive, and the titer was >1 : 32, so the diagnosis was confirmed for syphilis. Then the patient was treated with IM injection of 2.4 million units of benzathine penicillin. Following a week later for follow up, the papillary erosion scab desquamation and the erythema subsided, the bilateral palms and the soles of dark erythema also subsided. And the 2.4 million units of benzathine penicillin were administered intramuscularly once a week for three weeks totally. The patient is still in further follow-up.

## 3. Discussion

Syphilis is a sexually transmitted disease caused by *Treponema pallidum* [1]. The primary-stage of syphilis is mainly characterized by painless ulcers (chancre), which occur mostly in the genitals, anus, oral cavity [2], lips, pharyngeal, and nipple-areola [3]. There have been several reports of primary syphilis occurring on the nipple and, its clinical manifestations vary [4–7], such as erythema nodules on the nipple, swelling, erosion, indolent ulcers, asymptomatic scaly erythematous or crusted plaque, etc. This case is a unilateral nipple erythematous exudation change, which can be easily misdiagnosed as eczema, Paget's breast disease and so on. Histopathological and immunohistochemical results suggested that lymphocytes and plasma cells infiltrate the dermis, and the diseases of intradermal plasma cell infiltration are common in plasmacytosis, plasmacytoma, syphilis, and fungal infections. The dermis may have plasma cells, neutrophils, and lymphocytes infiltrated in primary syphilis. In immunohistochemistry Pax5, CD20 are expressed on the surface of B cells, CD3, are CD5 are the T cells markers. CD163 is expressed by the macrophages, CD38 is most strongly expressed in plasmocytes; however, Bc1-2(+), CD109(−), Bcl-6(−), MUM1(−), Cyclin D1(−), Alk(−), Ig*κ*(+), and Ig*λ*(+) could exclude the neoplastic lesion. The pathological results are consistent with clinical manifestations, highly indicating the diagnosis of syphilis. During the visit, the patient developed a typical second-stage syphilis change on the palm toe, which was characterized by a palm-toe rose rash. Further serological tests confirmed the final diagnosis. This patient was more unusual that there was an atypical syphilis and a typical second-stage syphilis during the visit. Due to no itchy erythema on skin or mucosa without related allergic history, it is necessary to consider the possibility of noncommon infection or tumor, thus pathological and serological examination are required.
